# Enhanced hybrid deep neural network for EEG-based schizophrenia diagnosis using functional and temporal features

**DOI:** 10.1038/s41598-025-26627-4

**Published:** 2025-11-28

**Authors:** Mahdi Soltani-Nejad, Farnaz Salar-pour, Seyed Ali Rakhshan, Hossein Nezamabadi-pour

**Affiliations:** 1 Intelligent Data Processing Laboratory (IDPL), Department of Electrical Engineering, Shahid Bahonar University of Kerman, Kerman, Iran; 2 Department of Electrical Engineering, Shahid Bahonar University of Kerman, Kerman, Iran; 3 Active Intelligent Systems (ACT) Laboratory, Southern University of Science and Technology (SUSTech), Shenzhen, China

**Keywords:** Hybrid deep learning, Schizophrenia, Classification, Machine learning, Diseases, Neurology

## Abstract

Schizophrenia is a complex psychiatric disorder that disrupts cognition, emotions, and social behavior. Timely and accurate diagnosis is essential for effective treatment. Traditional diagnostic methods relying on clinical assessments have limitations, including subjectivity and time consumption. To address these challenges, there is increasing interest in utilizing neuroimaging techniques like electroencephalography (EEG) for schizophrenia diagnosis. EEG provides direct measures of brain activity and can reveal unique patterns associated with the disorder. This study proposes a novel approach that utilizes EEG signals to accurately diagnose schizophrenia, aiming to overcome the limitations of traditional methods. EEG data was collected from two groups: individuals diagnosed with schizophrenia and a healthy control group. During a visual task, EEG signals were recorded and underwent preprocessing to remove artifacts and noise. The data was segmented into non-overlapping time windows, and functional and time-domain features were extracted. These features were then used as inputs to a hybrid deep neural network specifically designed for EEG data. The primary objective of the network was to distinguish between healthy individuals and those with schizophrenia. The proposed approach was benchmarked against established methods such as support vector machines and k-nearest neighbors, and it demonstrated superior performance. The evaluation was carried out using a robust k-fold cross-validation approach. Various performance metrics, including accuracy, sensitivity, and characteristic criterion, were used to assess the diagnostic accuracy and discriminative power of the network. The results showed that the hybrid deep neural network effectively identified individuals with schizophrenia. This study highlights the potential of EEG-based diagnostic approaches in accurately diagnosing schizophrenia and offers a promising avenue for reducing subjectivity and improving the efficiency of the diagnostic process. Future research should focus on expanding the dataset, investigating generalizability across different populations, and exploring potential clinical applications in real-world settings.

## Introduction

### Background

Schizophrenia is among the most severe and disabling psychiatric disorders, profoundly affecting an individual’s well-being, social functioning, emotional regulation, and cognitive performance^1^. This complex condition typically manifests during the second or third decade of life, arising from an interplay of genetic vulnerability, environmental influences, and psychosocial stressors^2^. Early detection is essential; however, the absence of definitive biomarkers means that diagnosis largely depends on subjective clinical assessments^3^. Achieving an accurate diagnosis, therefore, requires an integrated approach that combines clinical evaluation with neuroimaging techniques. A range of brain imaging modalities–such as positron emission tomography (PET), magnetic resonance imaging (MRI), and electroencephalography (EEG)–provide valuable insights into brain function and are widely used in the investigation and diagnosis of various neurological and psychiatric conditions, including Alzheimer’s disease, autism, Parkinson’s disease, and schizophrenia^4–8^. Misdiagnosis of schizophrenia remains a major concern, as it can lead to inappropriate treatment strategies and, in severe cases, increased patient morbidity and mortality. Current diagnostic practices continue to suffer from limited accuracy and consistency, underscoring the urgent need for intelligent, data-driven methods to assist clinicians. Addressing this challenge, our study proposes a novel framework designed to achieve highly accurate and reliable detection of schizophrenia.

Beyond introducing the proposed method, this study also investigates strategies for selecting and integrating the most informative EEG features to further enhance diagnostic performance. Furthermore, given that schizophrenia shares overlapping symptoms with other neurological and psychiatric disorders, designing experiments capable of simultaneously detecting multiple mental health conditions represents a promising direction for future research. These contributions not only strengthen the immediate impact of our work but also lay the groundwork for broader advancements in neuropsychiatric diagnostics.

### Our work and main contributions

We propose a novel intelligent framework for schizophrenia diagnosis that simultaneously leverages two complementary types of features extracted from EEG data. Using recordings collected and preprocessed by our laboratory from patients with schizophrenia and healthy controls, followed by meticulous artifact removal and feature extraction, we demonstrate that our method achieves an accuracy of over 99%. Compared to conventional approaches such as k-nearest neighbors (KNN), support vector machines (SVM), convolutional neural networks (CNN), and MSSTNet, the proposed network consistently outperforms them across Accuracy, Specificity, Sensitivity, and AUC metrics.

### Research methodology

**Fig. 1:**
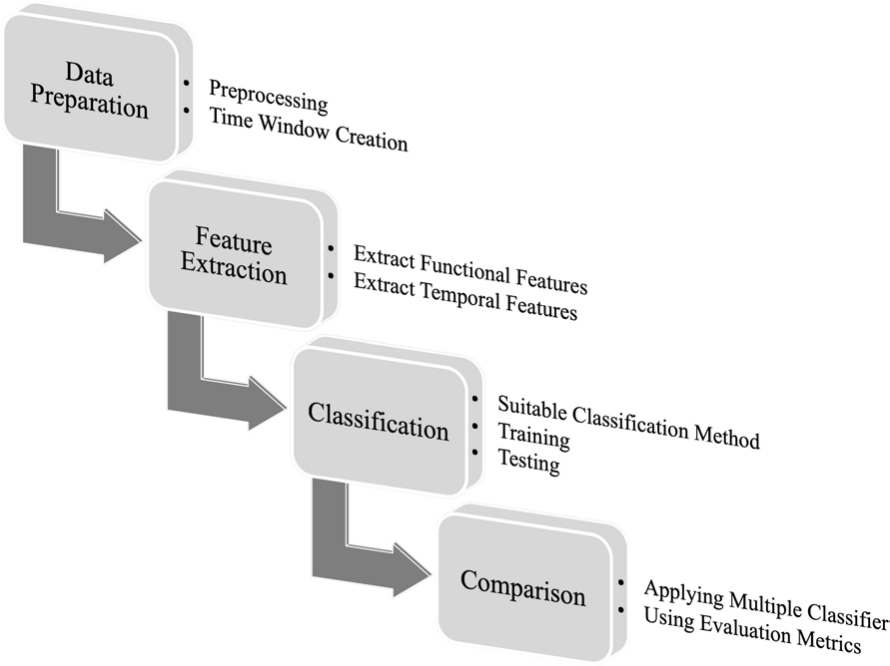
Main process of the paper.

Our methodology follows a systematic process in which EEG recordings are segmented into 25-second intervals to ensure consistent and reliable feature extraction. Rather than directly using raw EEG signals as classifier inputs, we extract two main categories of features: temporal features, which analyze each channel individually, and functional features, which capture the interactions between pairs of channels. These features are essential for representing the complex neural dynamics associated with schizophrenia. Figure 1 provides a schematic overview of the proposed methodology, including EEG signal acquisition, preprocessing, feature extraction, classification using the hybrid deep neural network, and comparison.

Traditional classifiers such as SVM and KNN rely on predefined feature sets and cannot efficiently process inputs with varying dimensions. In contrast, CNNs can handle structured feature sets but still face limitations when integrating heterogeneous feature types. To address this issue, we propose a neural network architecture with two separate input branches, allowing the model to effectively combine features of different dimensions within a single framework. This approach eliminates the need to train multiple models for different feature types, enabling a more comprehensive representation of EEG characteristics.

Moreover, depending on the specific feature set used, the proposed architecture enables the design of a more efficient and specialized neural network structure, ultimately enhancing the overall classification performance. To rigorously assess the effectiveness of the proposed model, we evaluate its performance using four key metrics: accuracy, specificity, sensitivity, and area under the ROC curve (AUC).

### Literature review

Machine learning, a subfield of artificial intelligence, encompasses a diverse set of techniques for constructing data-driven models that uncover latent patterns and knowledge embedded within large datasets^9^. Recent studies have highlighted the potential of integrating machine learning with recurrent dynamic frameworks for complex biomedical analyses, such as infectious disease prediction^10,11^. Building upon these advancements, the present study employs advanced deep learning approaches for analyzing EEG signals to aid in the diagnosis of schizophrenia.

These models serve as abstract representations of the original data, developed through the extraction of relevant features. Machine learning algorithms automatically learn these representations from the training data and subsequently apply them to predict or classify unseen test samples. For instance, long short-term memory (LSTM) recurrent neural networks (RNNs) have been used for diagnostic classification based on pediatric intensive care unit data^12^. Similarly, RNNs and Bayesian frameworks have been utilized to differentiate patients with ovarian cancer^13,14^, while support vector machines (SVMs) have been applied to predict attention deficit hyperactivity disorder (ADHD)^15^. In parallel, convolutional neural networks (CNNs) have been employed for EEG-based emotion recognition^16^, and hybrid architectures combining multilayer perceptrons (MLPs) and SVMs have been proposed for diagnosing major depressive disorder^17^.

In the context of schizophrenia classification using EEG data, machine learning techniques have been extensively investigated^18^. These approaches can be broadly categorized based on three key aspects: the type of algorithm employed, the characteristics of the extracted features, and the methodology used for EEG signal acquisition and preprocessing^19^. Table 1 summarizes several representative studies in this field.

**Table 1:** Comparison of machine learning techniques for schizophrenia diagnosis.

Study	Methodology	Features	Accuracy
Phang et al.^20^	DNN with DBN	Connectivity measures	Significant
Phang et al.^21^	CNN	Multi-domain features	Enhanced
Zhang^22^	Random forest	N1 and P3 Features	81%
Ahmedt et al.^23^	CNN	ERPs	72%
Li et al.^24^	ANN	Power Spectrum	Varied
Alimardani et al.^25^	KNN	SSVEP Features	Higher Performance

Phang et al.^20^ introduced a novel framework for automatic schizophrenia classification using EEG effective connectivity. Their method employed a deep neural network with a deep belief network (DBN) architecture, extracting features through vector autoregression–based directed connectivity and graph-theoretical network measures. Applied to a large resting-state EEG dataset, the approach revealed a marked reduction in neural oscillation synchronization (as measured by partial directed coherence) and diminished network integration (weighted degrees and transitivity) in individuals with schizophrenia compared to healthy controls. In a subsequent study, Phang et al.^21^ developed a multi-domain connectome CNN (MDC-CNN) designed to capture diverse aspects of disrupted connectivity. This model integrated time- and frequency-domain measures of effective connectivity with complex topological features, employing parallel 1D and 2D CNNs and fusion strategies to combine information across domains.

In another study, Zhang^22^ employed a random forest algorithm to distinguish schizophrenia patients from healthy controls based on N1 and P3 features derived from basic sensory tasks involving auditory tones and button presses, achieving 81% accuracy. Ahmedt et al.^23^ compared KNN, decision tree, and SVM classifiers for detecting schizophrenia risk in children using mean amplitudes of early and mid-latency event-related potentials from a passive auditory task. While traditional classifiers achieved only 44% accuracy, the performance improved significantly to 72% when a CNN-based deep learning approach was applied. Li et al.^24^ investigated artificial neural networks (ANNs) to classify EEG signals from patients with depression, schizophrenia, and healthy individuals, focusing on power spectrum features. Their study explored both backpropagation-based ANNs and self-organizing competitive ANNs. Alimardani et al.^25^ examined steady-state visual evoked potentials (SSVEP) induced by frequency-modulated visual stimuli for schizophrenia detection. Multiple classifiers were tested, with KNN combined with Fisher-scored features yielding superior performance relative to other methods. In more recent work, graph signal processing and graph learning approaches have also been applied to EEG data to model brain connectivity and extract discriminative patterns for schizophrenia identification^26^. Beyond psychiatric disorder detection, EEG-based deep learning has also been explored for cognitive state monitoring. For instance, MSSTNet has been proposed as a multi-stream time-distributed spatio-temporal deep learning framework that effectively detects mind wandering from EEG signals^27^.

Table 1 summarizes these machine learning techniques, highlighting their methodologies, feature choices, and classification accuracies. A broader survey of related studies is provided in Table 2.

Based on the comparative overview in Table 2, it can be observed that most existing studies on EEG-based schizophrenia detection have relied either on temporal/frequency-domain features (e.g., wavelet transforms, scalograms, RNN-LSTM models) or on functional connectivity features (e.g., GCNs, 3D-CNNs on brain connectivity, late fusion of graph indices). While some attempts at feature fusion exist, none of the reviewed works have systematically integrated both temporal dynamics and functional connectivity features simultaneously in a unified framework. This limitation restricts the models from fully capturing the complex spatio–temporal patterns underlying schizophrenia-related brain activity.

In our work, we address this gap by proposing a novel method that simultaneously leverages temporal and functional EEG features. Experimental results demonstrate that our approach achieves over 99% accuracy in schizophrenia detection, significantly outperforming previous state-of-the-art methods.

**Table 2:** Survey of EEG signal processing and deep learning models in schizophrenia detection.

Dataset / subjects	EEG features	Method / model	Evaluation metrics	Reported results / findings	Refs
EEG SZ vs HC (Sci Rep)	Scalogram / image-encoded EEG	Transfer learning (pretrained CNNs) + wrapper feature selection	Accuracy	Three-stage process: signal image encoding, feature extraction with pretrained models, wrapper feature selection; reported improved SZ detection accuracy	^1^
Graph-modeled EEG	3D functional connectivity features	3D-AGCN	Accuracy, AUC	Adaptive graph-based model captured spatio-temporal dependencies; showed significantly improved SZ classification performance	^28^
Multi-channel EEG dataset	Raw multichannel EEG signals	CNN-based deep learning model	Accuracy, Sensitivity, Specificity	CNN demonstrated effective feature extraction from raw EEG and achieved high diagnostic accuracy	^29^
Multichannel EEG SZ/HC	MIMFs (from MEMD) + spectral indices	Crossover-boosted Archimedes Optimization + Rough Sets	Accuracy	Optimization-based feature selection enhanced SZ detection accuracy	^30^
Not specified	Channel selection and feature fusion from EEG	Channel selection + fusion algorithm	Accuracy, Specificity, Sensitivity, AUC	Proposed an “afloat” channel selection and fusion strategy that improved classification performance for schizophrenia detection	^31^
Resting-state EEG SZ/HC	EEG microstates (A–D)	Deep CNN	Accuracy, AUC	Microstates classified with DCNN; potential biomarkers for SZ identified	^32^
EEG SZ/HC	Dynamic functional connectivity	3D-CNN	Accuracy	Combined connectivity dynamics with 3D-CNN achieved high SZ classification accuracy	^33^
EEG SZ/HC	Six connectivity indices	Late fusion deep learning	Accuracy	Fusion of multimodal connectivity indices produced strong SZ detection results	^34^
Multichannel EEG SZ/HC	Optimized preprocessed signals	Mutation-boosted Archimedes Optimization + deep learning	Accuracy	Optimized preprocessing pipeline significantly improved classification performance	^35^
EEG SZ/HC	Scalogram (CWT)	CNN / transfer learning	Accuracy	Scalogram time–frequency features provided discriminative information and high SZ accuracy	^36^
45 SZ, 39 HC	Raw/time-series EEG	LSTM + Dense layers	Accuracy ($\sim$98%)	RNN-LSTM achieved very high detection accuracy for SZ	^37^
EEG SZ/HC	(1) DWT / Scalogram, (2) Wavelet spectral	(1) CNN / transfer, (2) Wavelet autoencoder + EfficientNet	Accuracy	Exact paper not found; closest studies show strong performance using DWT/CWT + deep learning (scalograms, wavelet-autoencoders).	^38^
EEG recordings	Frequency-domain features + connectivity-based features	Fusion approach with ML/DL classifiers	Accuracy, Specificity, Sensitivity, AUC	Feature fusion of frequency and connectivity information led to improved diagnostic performance	^39^
Emotional EEG datasets	Emotional EEG features	Lightweight LLM pipeline	Accuracy (emotion recognition)	Not SZ-related; focused on affective EEG interpretation and automated report generation	^40^
Multi-disease EEG	Graph-neighborhood channel features	Graph Capsule Network (TriCaps)	Accuracy	General framework for multi-disease EEG classification, not specific to SZ	^41^
EEG SZ/HC	Hidden representations of 3D-CNN	3D-CNN + hidden-layer aggregation	Accuracy, Interpretability	Framework improved SZ screening and interpretability of learned EEG features	^42^
EEG datasets incl. SZ	Sparse, transfer, and hybrid features	Sparse + Transfer + Deep learning	Accuracy	Hybrid feature model applied to EEG, including SZ datasets.	^43^
EEG data modeled as graphs	Neural connectivity features (graph-based)	Hybrid GCN + LSTM model	Accuracy, AUC	Achieved strong performance by capturing spatial–temporal dependencies in EEG data	^44^
Resting-state EEG SZ/HC	EEG microstates	Deep CNN	Accuracy, Inference time	Achieved high accuracy in SZ detection using microstates with short processing time.	^45^
Multi-disorder EEG	Multi-domain features	Fuzzy logic + Spiking NN	Accuracy	Multi-disease EEG diagnosis; applicable to SZ but not SZ-specific	^46^
EEG signals from SZ patients & controls	Ratio indices derived from EEG bands (e.g., theta/alpha, delta/beta)	Statistical + ML methods	Accuracy	Ratio-based indices from EEG brainwaves provided discriminative features for distinguishing SZ patients from healthy controls	^47^
EEG SZ/HC	Learned + fuzzy-type features	CNN with fuzzy type-2 activations	Accuracy	Fuzzy DL model reduced uncertainty in EEG and improved SZ classification	^48^
EEG Depression patients	Multiband EEG features	Classical ML + DL	Accuracy	Focused on MDD (not SZ); included here since in user’s list	^49^

## Results

### Schizophrenia dataset

EEG data were collected in a controlled laboratory environment, with participants seated comfortably in a chair. The dataset comprised 25 recordings from healthy control subjects and 26 recordings from individuals diagnosed with schizophrenia. Participants were between 18 and 45 years of age.

To capture the EEG signals, a cap montage with 31 active electrodes was used, following the 10-20 system. The electrode positions included FP1, FP2, F7, F3, Fz, F4, F8, FT9, FC5, FC1, FC2, FC6, FT10, T7, C3, C4, T8, TP9, CP5, CP1, CP2, CP6, TP10, P7, P3, Pz, P4, P8, O1, Oz, and O2. Additionally, one terrestrial channel was included, resulting in a total of 32 channels.

During data acquisition, EEG signals were sampled at 500 Hz. The amplifier gain was adjusted to maintain a signal output-to-input ratio of 50. To filter out local electrical interference, waves in the frequency range of 45 to 55 Hz were eliminated using a notch filter. To prepare the EEG data for classification and minimize amplitude-related variability across conditions (i.e., between schizophrenia patients and healthy controls, as well as across the star and triangle recognition tasks), z-score normalization was applied. This procedure standardized amplitude ranges across all participants and experimental conditions, ensuring that amplitude differences did not confound feature extraction or classification performance.

Features such as the Phase Lag Index (PLI), Phase Locking Value (PLV), and Pearson Correlation Coefficient (Pearson CC) are inherently robust to amplitude fluctuations, as they quantify phase synchronization or correlation patterns rather than absolute amplitudes. In contrast, features such as Variance and Absolute Energy are directly influenced by amplitude dynamics, potentially reflecting neurophysiological differences between schizophrenia and healthy groups.

**Fig. 2:**
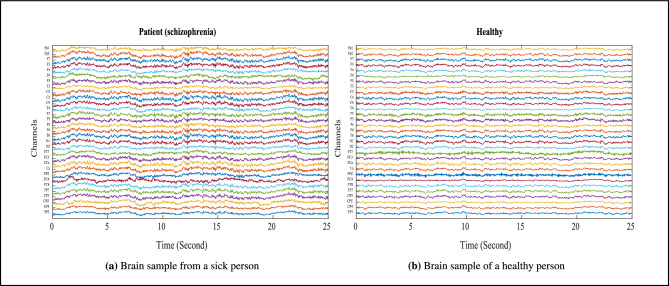
EEG signals.

The EEG signals were collected while the participants performed three tests: the star, circle, and recognition of triangle tests. The entire recording session lasted for 16 minutes, with 10 minutes (600 seconds) of active EEG recording per participant, divided into four blocks with 2-minute rest periods between them. Each 10-minute EEG recording was segmented into 25-second time windows, yielding 24 windows per participant ($600 \div 25 = 24$). With 51 participants (26 schizophrenia patients and 25 healthy controls), this resulted in a total of 1224 time windows. After removing windows contaminated by artifacts and noise, the final dataset comprised 803 time windows (409 for schizophrenia, 394 for healthy controls), which were used for feature extraction and classification. Figure 2 depict sample EEG signals from a person diagnosed with schizophrenia and a healthy individual, respectively.

### Machine learning results

**Table 3:** Hyperparameter configurations of baseline and proposed methods.

Classifier	Hyperparameter	Values considered	Selected value	Optimization method
KNN	Number of neighbors (k)	{1, 5, 10, 15, 20, 25, 30}	25	Manual
	Distance metric	{Euclidean, Cityblock, Cosine}	Cityblock	Manual
	Search method	{Exhaustive, KDTree}	Exhaustive	Fixed
	Standardization	{Yes, No}	No	Fixed
SVM	Kernel type	{Linear, RBF, Polynomial}	RBF	Auto
	Kernel scale ($\gamma$)	{0.01, 0.1, 1, auto}	Auto	Default
	Standardization	{Yes, No}	Yes	Fixed
	Outlier fraction	{0, 0.01, 0.05, 0.1}	0.05	Manual
CNN	Input size	[1$\times$31$\times$1]	Fixed	–
	Convolutional layers	{1–4}	3 (filters=8,16,32; size=3$\times$3)	Manual
	Batch normalization	After each conv layer	Applied	Fixed
	Activation	{ReLU, LeakyReLU}	ReLU	Fixed
	Dropout rate	{0.2, 0.3, 0.5}	0.3	Manual
	Optimizer	{SGD, Adam, RMSProp}	Adam	Manual
	Learning rate	{1e-2, 1e-3, 1e-4}	1e-3	Schedule
	Mini-batch / Epochs	{32, 64, 128} / {32, 64, 128}	128 / 64	Early stopping
MSSTNet classification	Input size	[h$\times$w$\times$c], numFeatures=31	From STFT (spectrogram)	Fixed
	CNN filters	{8, 16, 32}	16	Manual
	Filter size	{3$\times$3, 5$\times$5}	3$\times$3	Manual
	RNN hidden units	{50, 100, 150}	100	Manual
	Concatenation	CNN + RNN (bilinear pooling)	Applied	–
	Optimizer	{SGD, Adam, RMSProp}	Adam (lr=1e-4)	Manual
	Learning rate	{1e-2, 1e-3, 1e-4}	1e-4	Fixed
	Mini-batch size	{16, 32, 64}	32	Manual
	Epochs	{100, 200, 300}	200 (early stop, patience=10)	Early stopping
Proposed method	Input size	[h$\times$w$\times$c], numFeatures=31	Fixed	–
	CNN filters	{8, 16}	8, 16	Manual
	Filter size	{3$\times$3, 5$\times$5}	3$\times$3	Manual
	BiLSTM hidden units	{50, 100, 150}	100 (1st), 50 (2nd)	Manual
	Concatenation	CNN + BiLSTM features	Applied	–
	Optimizer	{SGD, Adam}	Adam	Manual
	Learning rate	{1e-2, 1e-3, 1e-4}	1e-3	Schedule
	Mini-batch size	{16, 32, 64}	32	Manual
	Epochs	{50, 70, 100}	70	Early stopping

**Table 4:** Feature selection and its impact on performance metrics (accuracy, sensitivity, specificity, AUC).

Functional	Temporal	Accuracy (%)	Sensitivity (%)	Specificity (%)	AUC (%)
Pearson correlation coefficient	All	97.50	96.51	98.64	97.58
Coherence	All	81.87	80.12	84.61	82.30
PLI	All	96.25	96.51	95.94	96.22
PLV	All	97.50	94.93	99.10	97.46
Pearson correlation coefficient PLV	All	97.50	98.83	95.94	97.39
Coherence PLV	All	89.75	91.50	86.72	89.87
PLI PLV	All	98.125	98.91	97.05	97.98
PLI PLV Pearson correlation coefficient	All	98.125	99.20	96.05	98.02
PLI PLV coherence	All	96.25	97.43	95.12	96.27
All	All	97.50	96.38	98.70	97.54
PLI PLV Pearson correlation coefficient	All	98.75	98.78	98.71	98.70
PLI PLV Pearson correlation coefficient	All	98.75	98.780	98.71	98.70
PLI PLV Pearson correlation coefficient	All	98.75	98.78	98.71	98.70
PLI PLV Pearson correlation coefficient	Shannon entropy	99.37	98.71	99.3	99.35
PLI PLV Pearson correlation coefficient	Hjorth mobility	98.12	95.89	99.10	97.94
PLI PLV Pearson correlation coefficient	Variance absolute energy	96.87	95.89	97.70	96.79
PLI PLV Pearson correlation coefficient	Absolute energy auto regression	98.75	98.92	98.50	98.71
PLI PLV Pearson correlation coefficient	Shannon entropy absolute energy	99.31	99.39	98.76	99.38
PLI PLV Pearson correlation coefficient	Hjorth mobility absolute energy	99.37	99.14	98.78	99.39
PLI PLV Pearson correlation coefficient	Variance absolute energy Hjorth mobility	98.12	96.77	99.15	98.38
PLI PLV Pearson correlation coefficient	Absolute energy auto regression Hjorth mobility	98.125	98.76	97.46	98.11
PLI PLV Pearson correlation coefficient	Shannon entropy absolute energy Hjorth mobility	97.56	98.90	95.65	97.27
** PLI** **PLV** **Pearson correlation coefficient**	** Variance** **absolute energy** **auto regressionHjorth mobility**	**99.12**	**99.03**	**99.24**	**99.13**
PLI PLV Pearson correlation coefficient	Absolute energy auto regression Shannon entropy Hjorth MOBILITy	98.12	98.87	97.18	98.00
PLI PLV Pearson correlation coefficient	All	98.75	98.78	98.71	98.70

Significant values are in bold

**Fig. 3:**
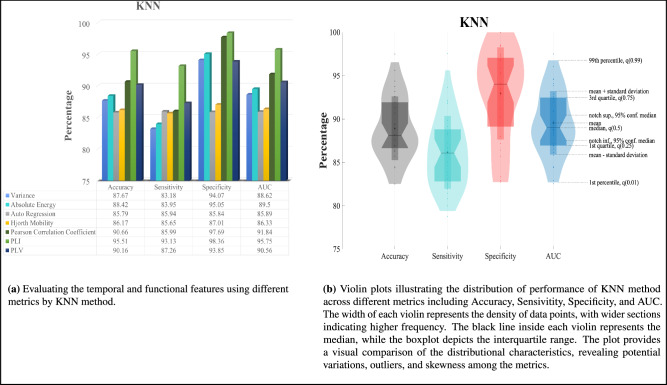
KNN classification results.

**Fig. 4:**
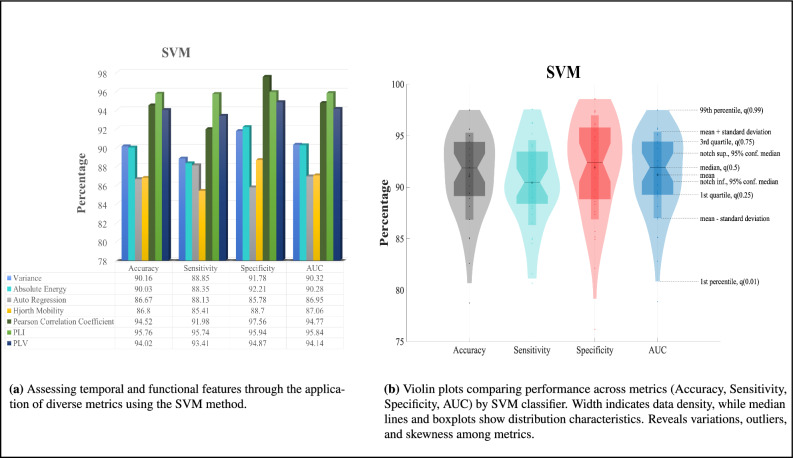
SVM classification results.

**Fig. 5:**
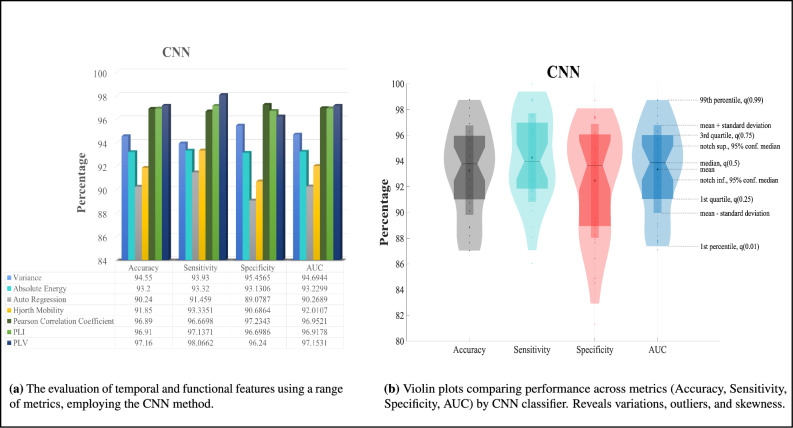
CNN classification results.

**Fig. 6:**
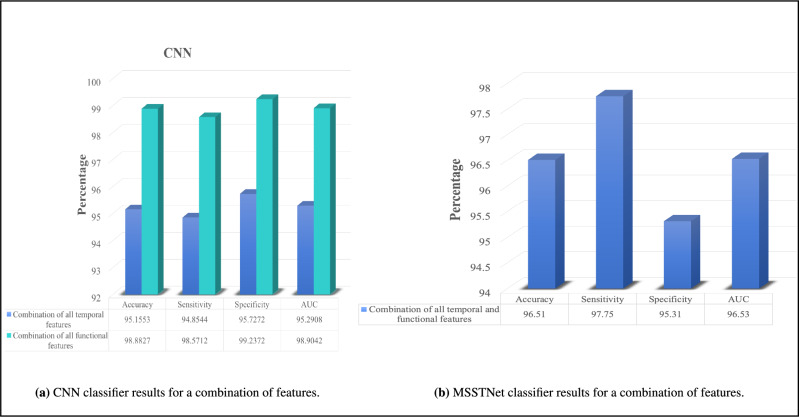
Classification results for combination features.

We have provided the specifications of the employed methods in Table 3. Figures 3, 4, 5, 6 present the major scores obtained for KNN classification, SVM classification, CNN classification, and MSSTNet Classification respectively. The evaluation was performed separately for each temporal feature, including Variance, Absolute Energy, Auto Regression, and Hjorth Mobility, as well as for each functional feature, namely Pearson correlation coefficient, PLI, and PLV. Accuracy, Sensitivity, Specificity, and AUC were utilized as metrics to assess the classification method’s performance.

As shown in Fig. 3a, when evaluating the temporal features using the accuracy metric, the KNN method demonstrates superior performance in disease diagnosis specifically with the Absolute Energy feature. Similarly, among the functional features, the KNN method performs better in diagnosing schizophrenia disorder from EEG signals, particularly with the PLI feature. Overall, Fig. 3a illustrates that the KNN method outperforms other methods in diagnosing schizophrenia disorder using the Absolute Energy and PLI features, as indicated by various performance metrics. However, it is important to note that in terms of the sensitivity metric, the KNN method with Auto Regression feature demonstrates better performance in disease diagnosis. in addition, we employ violin plots in Fig. 3b to visually analyze the distribution of performance metrics, including Accuracy, Sensitivity, Specificity, and AUC, revealing variations, outliers, and skewness among these metrics. The ROC curve of kNN method is illustrated in Fig. 9a.

As shown in Fig. 4a, the SVM method demonstrates superior performance in disease diagnosis among the temporal features, specifically with the Variance feature. This is evident from the results of all metrics except for specificity. However, when considering specificity, the SVM method performs better when utilizing the Absolute Energy feature. The scores obtained for the temporal features range from around 88% to 93% using the SVM method. Similarly, among the functional features, the SVM method outperforms with the PLI feature in terms of accuracy, sensitivity, and AUC metrics. The scores indicate that the SVM method achieves better results in disease diagnosis when utilizing the PLI feature. However, when considering the specificity metric, the Pearson correlation coefficient feature yields better results. The scores obtained for the functional features range from around 95% to 98% using the SVM method. In addition, we utilize violin plots in Fig. 4b to investigate the distribution patterns of performance metrics, such as Accuracy, Sensitivity, Specificity, and AUC. The ROC curve of SVM method is illustrated in Fig. 9b.

As depicted in Fig. 5a, the CNN method exhibits superior performance in disease diagnosis among the temporal features, specifically with the Variance feature. The scores obtained for the temporal features range from approximately 93% to 96% when employing the CNN method. Similarly, among the functional features, the CNN method outperforms with the PLV feature in terms of accuracy, sensitivity, and AUC metrics. The scores suggest that the CNN method achieves better results in disease diagnosis when utilizing the PLV feature. However, when considering the specificity metric, the Pearson correlation coefficient feature yields better results. The scores obtained for the functional features range from around 97% to 99% when using the CNN method. In addition, the utilization of violin plots in Fig. 5b for CNN method provides a rich visual representation that facilitates the exploration and comparison of distributional characteristics among performance metrics, shedding light on potential variations and outliers.The ROC curve of CNN method is illustrated in Fig. 9c.

As previously mentioned, the CNN method is capable of accepting inputs in the form of high-dimensional matrices. Taking advantage of this capability, we combined the temporal features, creating a $31 \times 4$ matrix, and the functional features, creating a $465 \times 3$ matrix, separately. By doing so, we obtained the results of the CNN method. As illustrated in Fig. 6a, the results have significantly improved compared to Fig. 5a, indicating an enhanced performance of the method. Specifically, the combination of temporal features in diagnosing schizophrenia yields scores ranging from approximately 94.8% to 95.7%. Moreover, the CNN method demonstrates a substantial improvement in disease diagnosis when combining the functional features, resulting in scores between approximately 98.5% and 99.2%. The ROC curve of CNN method with the combination of temporal features and functional features, separately is illustrated in Fig. 9d. Figure 6b illustrates the MSSTNet method, which bears some similarity to our proposed approach. In this method, transfer learning is employed; therefore, parallel learning networks are not incorporated. As a result, the responses are comparatively less effective than those achieved by the method presented in this paper.

**Fig. 7:**
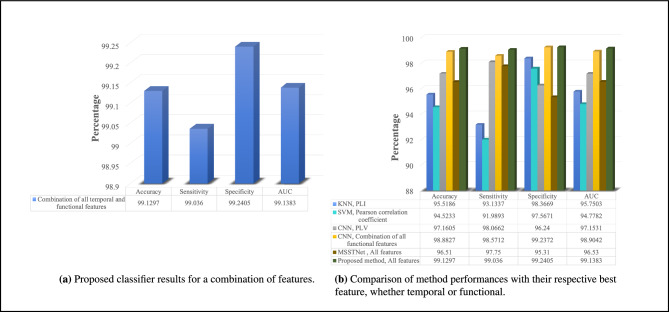
Comparison with BarPlots.

**Fig. 8:**
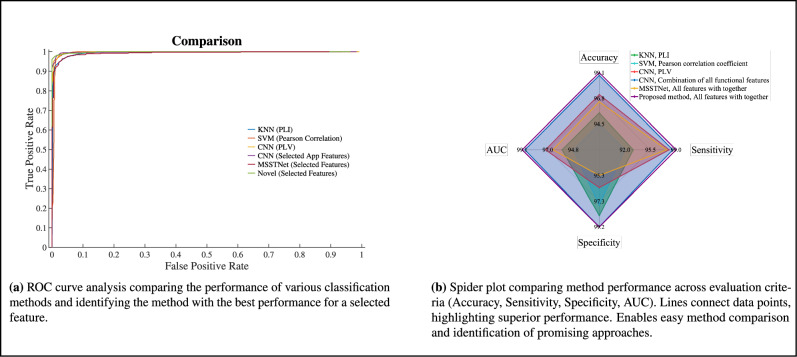
Comparison of all classification methods of this paper.

**Table 5:**
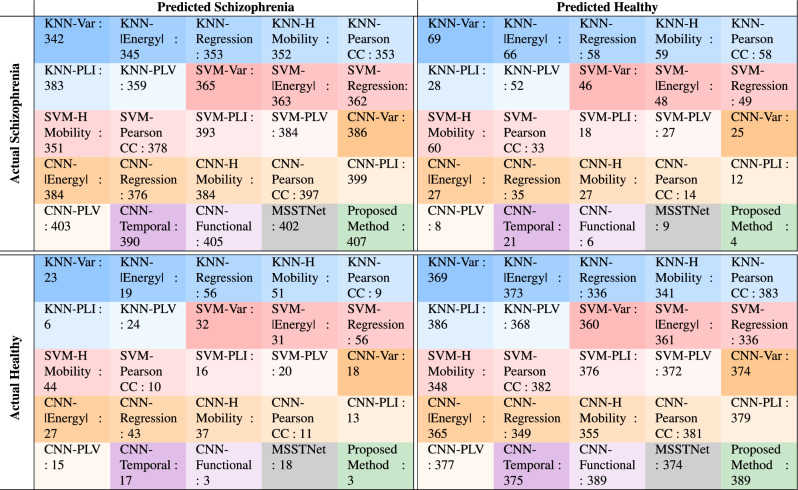
Confusion matrices for schizophrenia classification models.

To comprehensively evaluate the classification performance, Table 5 presents the confusion matrices for all methods and feature combinations used in this study. Based on 803 time windows (409 for schizophrenia, 394 for healthy controls), each matrix reports True Positives (TP, Actual Schizophrenia and Predicted Schizophrenia), True Negatives (TN, Actual Healthy and Predicted Healthy), False Positives (FP, Actual Healthy and Predicted Schizophrenia), and False Negatives (FN, Actual Schizophrenia and Predicted Healthy). The Proposed Method with the optimal feature combination (PLI, PLV, Pearson Correlation Coefficient, Variance, Absolute Energy, Auto Regression, Hjorth Mobility) achieves the lowest FN (4) and FP (3), demonstrating minimal misclassifications, which is critical for schizophrenia detection where missing true cases (FN) can have significant clinical implications. These matrices provide a detailed view of the classification performance across all methods and feature sets, complementing the results in Table 4.

To determine the optimal feature combination, we considered 4 functional features and 5 temporal features. A complete search of all possible combinations would require $2^{4}-1 = 15$ combinations of functional features and $2^{5}-1=31$ combinations of temporal features. Multiplying these values yields 465 possible combinations, making exhaustive evaluation computationally expensive and time-consuming. Therefore, we adopted the strategy presented in Table 4. In this approach, we first evaluated each individual functional feature in combination with all temporal features. Next, the best-performing functional feature was combined pairwise with the remaining functional features, and the evaluation was repeated iteratively. This process continued until the final step, where the optimal feature combination was identified, which is highlighted in bold in the table. In the remainder of this paper, whenever we refer to the “selected feature”, we specifically mean the bolded combination reported in Table 4.

**Fig. 9:**
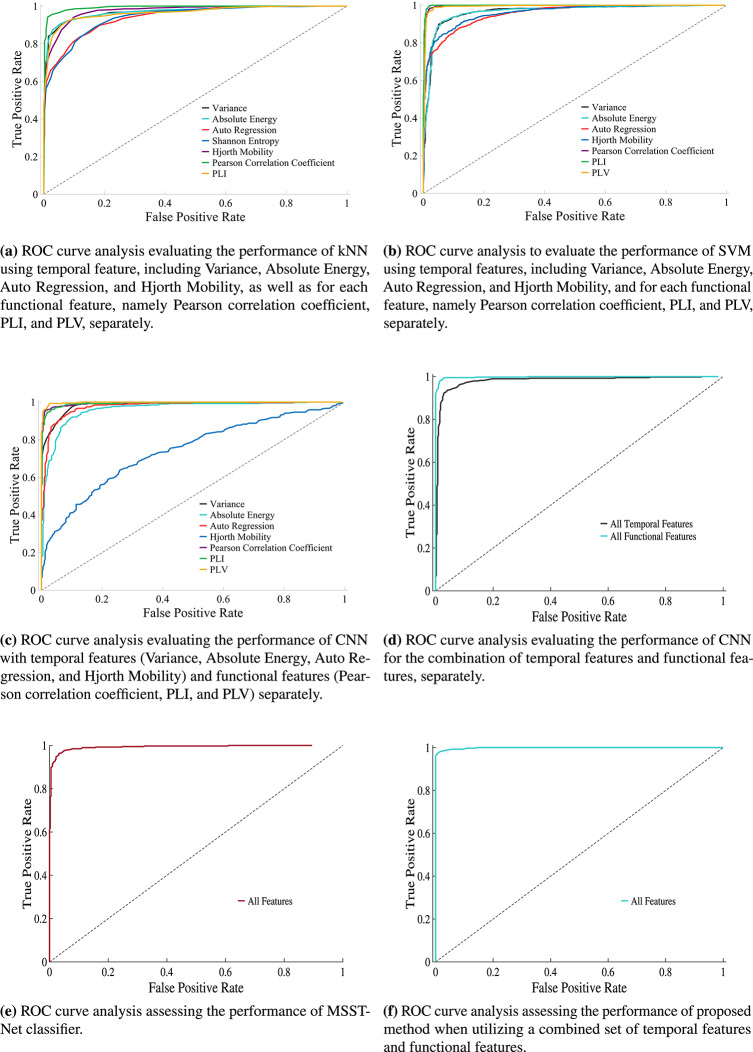
ROC plots.

In order to incorporate all temporal and functional features simultaneously, while preserving the unique properties of each feature category, traditional methods such as KNN, SVM or CNN are not suitable. Even the MSSTNet method is not suitable for our work, since it does not involve comprehensive learning and only the fully connected layers are trained. This approach is more appropriate for applications that directly utilize EEG data and do not require feature extraction. Therefore, as described in Section Methods of the article, we employed the proposed combined method. As demonstrated in Fig. 7a, the results have experienced a significant improvement, leading to an excellent diagnosis score for schizophrenia. The scores for all metrics range between 99% and 99.3%. The ROC curve of proposed method is illustrated in Fig. 9f.

For a concise comparison, it is recommended to refer to Fig. 7b, where a summary of the results from Figs. 3,4,5, and 6 is presented. Each method is accompanied by its best performance using one of the features, whether it is a temporal or functional feature. It can be observed that among all the results, the proposed method in this article yields the most impressive outcomes. In addition, we utilize a spider plot in Fig. 8b to visually examine the performance profiles of different methods across multiple evaluation criteria, including Accuracy, Sensitivity, Specificity, and AUC. This plot effectively summarizes the strengths and weaknesses of each method, facilitating straightforward comparisons and the identification of the most promising approaches. The ROC curve of all method with the best performance for a selected feature is illustrated in Fig. 8a.

## Discussion

In this study, we conducted a comprehensive evaluation of multiple classification methods for diagnosing schizophrenia based on EEG signals, focusing on the performance of these methods when incorporating both temporal and functional features extracted from the data.

We first assessed traditional classifiers, including KNN, SVM, and CNN, using temporal features. The KNN method yielded promising results, particularly with the Absolute Energy feature. In contrast, the SVM classifier achieved superior performance with the Variance feature, except for specificity, where the Absolute Energy feature performed slightly better. The CNN model, well-suited for handling high-dimensional data, demonstrated excellent results with the Variance feature. Notably, integrating all temporal features substantially improved diagnostic accuracy, especially for the CNN classifier.

For functional features, the KNN method performed best with the PLI, while the SVM achieved higher accuracy with the PLV. Interestingly, when functional features were evaluated individually, the CNN also achieved strong results with the PLV feature. However, combining all functional features further enhanced diagnostic scores, confirming the advantage of integrating complementary information.

The proposed combined method, which integrates both temporal and functional features while maintaining their intrinsic characteristics, emerged as the most effective approach. It achieved diagnostic scores ranging from 99% to 99.3% across all evaluation metrics, demonstrating exceptional performance. This outcome highlights the power of leveraging the complementary nature of temporal and functional information, leading to significantly improved classification accuracy over individual feature sets.

In summary, this study emphasizes the importance of jointly considering temporal and functional EEG features in the automated diagnosis of schizophrenia. The proposed integrated framework outperformed individual classifiers, offering a reliable and accurate diagnostic tool. These findings have important implications for improving the precision of schizophrenia diagnosis and for guiding the design of future intelligent diagnostic systems. Validation of this framework on larger and more diverse datasets will be essential to confirm its robustness and clinical applicability.

Future research can extend this work in several directions. One promising avenue is the systematic identification and selection of the most informative temporal and functional features, alongside optimization of feature fusion strategies to further enhance diagnostic reliability. Additionally, designing experiments that enable the simultaneous detection of multiple neurological and psychiatric disorders is crucial, given the substantial overlap in symptoms among these conditions. Expanding the framework to larger, multi-center datasets would also improve its generalizability and support its translation into real-world clinical environments.

## Methods

This section describes the methodology for processing EEG signals to diagnose schizophrenia using a hybrid deep neural network. The pipeline is presented step-by-step, covering data acquisition, preprocessing, feature extraction, and integration into the proposed model architecture, with mathematical formulations to ensure clarity and reproducibility.

### EEG signal acquisition

EEG data were collected from 51 participants (25 healthy controls and 26 schizophrenia patients, diagnosed per DSM-IV criteria) aged 18–45 years, matched for age, gender, and education. Inclusion criteria included right-handedness (assessed via the Edinburgh Handedness Inventory). Exclusion criteria were: (a) first-degree relatives with schizophrenia (for controls), (b) recent electroconvulsive therapy (within 6 months, for patients), (c) neurological disorders (e.g., epilepsy, head trauma, stroke), and (d) substance abuse.

EEG signals were recorded using a 32-channel Mitsar-202 amplifier (Mitsar, Russia) at a sampling rate of 500 Hz, with a gain of 50, band-pass filtering at 0.16–30 Hz, and notch filtering at 45–55 Hz to remove line noise. A monopolar montage with 31 active electrodes (FP1, FP2, F7, F3, Fz, F4, F8, FT9, FC5, FC1, FC2, FC6, FT10, T7, C3, C4, T8, TP9, CP5, CP1, CP2, CP6, TP10, P7, P3, Pz, P4, P8, O1, Oz, O2) and one ground channel was used, with the reference set to the average of the left and right mastoids. The experimental task involved star and triangle stimuli presented in unilateral (25% right, 25% left) and bilateral (25% identical, 25% different) conditions, with 320 trials over 16 minutes, divided into four 4-minute blocks with 2-minute rest intervals. Each 10-minute active EEG recording was segmented into 25-second time windows, yielding 24 windows per participant ($600 \div 25 = 24$), resulting in 1224 windows across 51 participants. After artifact removal, 803 windows (409 schizophrenia, 394 healthy) were retained for analysis. Behavioral measures (error rate, reaction time) and ERP components (N100, P200, P300) were recorded, as detailed in Table 6.

**Table 6:** Demographic and Clinical Characteristics Of The Study Groups (Mean ± SD).

Variables	Patients (n = 26)	Controls (n = 25)	p-value
Age (years)	$33.8 \pm 8.2$	$34.4 \pm 8.8$	0.7
Male (%)	$20\,(80\%)$	$20\,(77\%)$	0.8
Education (years)	$11.2 \pm 2.4$	$11.7 \pm 1.2$	0.6
Right-handed (%)	$89.3 \pm 5.4$	$98.5 \pm 6.4$	0.7
Duration of illness (years)	$12.3 \pm 5.7$	–	–
Age at illness onset (years)	$21.4 \pm 5.9$	–	–
Chlorpromazine equivalent dose (mg)	$368 \pm 151$	–	–
Positive symptom score	$8.1 \pm 4.7$	–	–
Negative symptom score	$11.6 \pm 5.9$	–	–

During EEG signal acquisition, participants were instructed to minimize artifacts by avoiding blinking, muscle contractions, jaw clenching, and excessive movement. However, given the relatively long duration of the task, such artifacts were inevitable. Therefore, a preprocessing stage was performed to remove noise and artifacts, which is a critical step for ensuring reliable data. Preprocessing was conducted using EEGLAB (a MATLAB toolbox), which allows manual rejection of artifacts; in cases where correction was not feasible, contaminated segments were discarded. A band-pass filter (47–77 Hz) was applied to remove power-line noise, followed by high-pass and low-pass filtering (0.23–95 Hz) for further signal cleaning. Ocular artifacts, particularly those caused by eye blinks, were corrected using Independent Component Analysis (ICA). The components associated with eye blinks were identified in the frontal regions of the scalp and subsequently removed, resulting in artifact-free EEG signals suitable for further ERP analysis.

All experimental procedures involving human participants were conducted in accordance with relevant guidelines and regulations. The study protocol was reviewed and approved by the Institutional Ethics Committee of Shahid Bahonar University of Medical Sciences, Kerman, Iran. Informed consent was obtained from all participants prior to data collection, and all participants (or their legal guardians) voluntarily agreed to participate in the study. All EEG data were handled confidentially, and participants’ privacy and anonymity were fully preserved throughout the study.

### Data preprocessing

To ensure high-quality EEG data, preprocessing was performed using EEGLAB (MATLAB toolbox). The steps are as follows:

1. Segmentation: Raw EEG signals ($X \in \mathbb {R}^{C \times T}$, where $C = 32$ channels, $T = 500 \times 600 = 300,000$ samples for 10 minutes) were segmented into 25-second non-overlapping windows ($T_w = 500 \times 25 = 12,500$ samples), yielding $X_w \in \mathbb {R}^{32 \times 12,500}$ per window.
2. Filtering: A band-pass filter (0.23–95 Hz) removed low- and high-frequency noise, and a notch filter (47–77 Hz) eliminated power-line interference.
3. Artifact removal: Ocular and muscle artifacts were identified using Independent Component Analysis (ICA). Components associated with eye blinks (primarily in frontal channels) were removed. Contaminated windows were discarded, reducing the dataset to 803 windows.
4. Normalization: To standardize amplitude variations across participants and test conditions (star, circle, recognition triangle), z-score normalization was applied per channel within each window:
  $$X_w^{\text {norm}} = \frac{X_w - \mu _w}{\sigma _w},$$
  where $\mu _w$ and $\sigma _w$ are the mean and standard deviation of the signal $X_w$ for each channel over the 25-second window. This ensured robustness against amplitude differences while preserving phase and connectivity patterns relevant for functional features (e.g., PLI, PLV).

The preprocessed EEG signals ($X_w^{\text {norm}}$) were used for feature extraction and model input.

### Extracting features from EEG

In this study, a range of functional and time features were employed for analysis including nine different measures for analysing EEG data. The chosen time features included variance, absolute energy, auto regression, Shannon entropy, and mobility Hjorth. These measures were selected to encompass widely used metrics in both time and functional domains. In addition to the time features, functional features were also utilized. These included Pearson correlation coefficient, coherence, phase locking value, and phase lag index. These measures capture the relationship and synchronization between different electrode channels in the EEG signal.

We chose this range of measures because we aimed to include commonly used metrics in both the time and functional domains for our analysis. Now, let’s briefly explain the underlying theories behind each measure and the experimental setups we used to compute them in our study.

Hjorth parameters and the Phase Lag Index (PLI) were employed as EEG features. The rationale behind this selection is that these measures capture fundamental neurophysiological characteristics that are particularly relevant to schizophrenia. Hjorth mobility reflects the signal’s mean frequency and provides insight into brain dynamics, which are often altered in patients with schizophrenia. The PLI, on the other hand, is a robust measure of phase synchronization between EEG channels that is insensitive to volume conduction, making it suitable for detecting abnormal functional connectivity patterns. Previous studies have reported that both Hjorth parameters and PLI show significant discriminative ability between patients with schizophrenia and healthy controls, thus supporting their suitability as input features for classification models.

Two types of features were extracted from each 25-second window to capture temporal and functional characteristics of EEG signals:

1. Temporal features: these characterize individual channel dynamics:
  Variance: variance is a statistical metric employed to quantify the dispersion or range among numbers within a dataset. It provides insights into the interrelationship of individual values. Variance is commonly used alongside other measures like standard deviation and covariance to gain a comprehensive understanding of the data. The variance is computed using the following formula:
    $$\text {Var}(X_w^{\text {norm}}) = \frac{1}{T_w} \sum _{t=1}^{T_w} (X_w^{\text {norm}}(t) - \mu _w)^2,$$
    where $\mu _w= \frac{1}{T_w} \sum _{t=1}^{T_w} X_w^{\text {norm}}(t)$ is the mean of the data points. The variance represents the average of the squared differences between each data point and the mean of the signal. A higher variance indicates greater variability or fluctuations in the EEG signal, while a lower variance suggests more stability or consistency^50,51^. Statistical analysis and machine learning algorithms can be utilized to compare the variances of EEG signals among different groups, such as individuals with schizophrenia. By examining these variations, specific patterns or characteristics associated with schizophrenia can potentially be identified.
  Absolute energy: absolute energy is a time-domain feature commonly used in the analysis of EEG signals^52^. It measures the overall signal strength or magnitude by summing the squared amplitudes of the signal values. Absolute energy provides insights into the intensity or power of the EEG signal. The absolute energy is determined by applying the following formula:
    $$E(X_w^{\text {norm}}) = \sum _{t=1}^{T_w} |X_w^{\text {norm}}(t)|^2.$$
    The absolute value of each data point is squared and then summed over the entire signal duration. This calculation effectively squares the amplitudes, ensuring that both positive and negative values contribute to the energy calculation. In EEG analysis, absolute energy serves as a valuable feature for evaluating the overall power and intensity of brain activity. It allows us to compare energy levels across different frequency bands or brain regions. Variations in absolute energy can indicate changes in neural activity, such as heightened or reduced brain activation. By comparing the absolute energy of EEG signals from different groups, statistical analysis or machine learning algorithms can potentially uncover energy-related patterns or abnormalities associated with schizophrenia.
  Auto regression (AR): autoregressive models utilize regression techniques on lagged series derived from the original time series. In multiple linear regression, the output is determined by a linear combination of several input variables. In the context of autoregression models, the output represents a future data point and can be expressed as a linear combination of previous p data points, where p denotes the lag window^53^. The autoregression model can be represented by the following equation:
    $$X_w^{\text {norm}}(t) = c + \sum _{i=1}^p \phi _i X_w^{\text {norm}}(t-i) + \epsilon _t,$$
    Here, $c$ represents a constant term, $\phi _i$ are the autoregressive coefficients, and $\epsilon _t$ denotes the error term or residual at time $t$. The autoregressive coefficients capture the relationship between the current value and the past values of the signal. Autoregressive modeling is essential in EEG analysis as it captures the temporal patterns and dynamics in the brain’s electrical activity. By estimating autoregressive coefficients, researchers can reveal underlying processes and dependencies in EEG data. This modeling technique enhances the ability to predict future signal values and identify abnormalities, resulting in more accurate diagnoses and effective treatment strategies in EEG-based neurology.
  Shannon entropy: the Shannon entropy is a widely used statistical measure that helps characterize complex processes. It is particularly effective in detecting nonlinearity in model series, providing a more accurate understanding of the nonlinear dynamics at various points of analysis. This, in turn, improves our comprehension of complex systems characterized by complexity and nonequilibrium, allowing for a more reliable explanation of their nature^54^. The Shannon entropy is calculated using the following formula:
    $$H(X_w^{\text {norm}}) = -\sum _{i=1}^K p_i \log _2(p_i),$$
    Here, $K$ represents the number of unique values in the sequence, and $p_i$ is the probability of occurrence of the ith value. To calculate entropy, the probabilities of each unique value in a sequence are computed, and the logarithm of these probabilities is taken. The negative sign is applied to ensure that the resulting entropy value is non-negative.
  Hjorth mobility: mobility is a feature derived from Hjorth parameters, which are statistical measures used to characterize the temporal dynamics of EEG signals. Hjorth parameters provide information about the signal’s activity, mobility, and complexity. Mobility specifically quantifies the frequency-dependent changes in the signal’s amplitude. To compute the mobility of an EEG signal, let’s assume we have a discrete sequence of N data points denoted as $x = [x_1, x_2, ..., x_N]$. The mobility (M) is calculated using the following formula:
    $$M(X_w^{\text {norm}}) = \sqrt{\frac{\text {Var}(\Delta X_w^{\text {norm}})}{\text {Var}(X_w^{\text {norm}})}},$$
    where $\Delta X_w^{\text {norm}} = [x_2 - x_1, x_3 - x_2, ..., x_N - x_{N-1}]$ is the first derivative of the signal. The Hjorth Mobility feature is widely used in EEG signal analysis due to its diverse applications. It offers valuable insights into the frequency-dependent variations in the amplitude of brain activity. By analyzing the mobility of EEG signals, researchers can delve into the dynamic properties of brain oscillations, identify abnormalities in neural function, and compare mobility characteristics across different brain regions or cognitive states. The Hjorth Mobility feature greatly contributes to enhancing our understanding of the temporal dynamics of EEG signals and their intricate relationship with brain function. It serves as a valuable tool for investigating brain activity and its underlying mechanisms^55^.
2. Functional features: these capture inter-channel connectivity:
  Pearson correlation coefficient (PCC): the Pearson correlation coefficient is a widely used measure of similarity between two variables. It is calculated as the covariance divided by the standard deviation. This coefficient is suitable for assessing the association between two variables measured on an interval or ratio scale which it ranges from -1 to 1, where -1 indicates a perfect negative linear relationship, 1 indicates a perfect positive linear relationship, and 0 indicates no linear relationship. However, it relies on certain assumptions, including a linear relationship between the variables and a bivariate normal distribution. When these assumptions are violated, such as in the case of non-linearity, the Pearson correlation coefficient becomes less robust. Even a single outlier can greatly influence the estimated value of the Pearson correlation coefficient^56^. The Pearson correlation coefficient (r) can be computed using the following formula:
    $$r_{ij} = \frac{\sum _{t=1}^{T_w} (X_{w,i}^{\text {norm}}(t) - \bar{X}_{w,i})(X_{w,j}^{\text {norm}}(t) - \bar{X}_{w,j})}{\sqrt{\sum _{t=1}^{T_w} (X_{w,i}^{\text {norm}}(t) - \bar{X}_{w,i})^2 \sum _{t=1}^{T_w} (X_{w,j}^{\text {norm}}(t) - \bar{X}_{w,j})^2}},$$
    Here, $\bar{X}_{w,i}$ and $\bar{Y}_{w,i}$ represent the mean values of the sequences $X_{w,i}^{\text {norm}}(t)$ and $Y_{w,i}^{\text {norm}}(t)$ respectively. The numerator of the formula computes the covariance between the two sequences, while the denominator normalizes the covariance by the standard deviations of $X_{w,i}^{\text {norm}}(t)$ and $Y_{w,i}^{\text {norm}}(t)$.
  Coherence: the concept of coherence holds significance in various fields including quantum physics, cosmology, physiology, brain research, and the study of consciousness. Coherence encompasses multiple definitions that are relevant to the investigation of human physiology, social interactions, and global affairs. The common dictionary definition of coherence refers to the quality of being logically integrated, consistent, and understandable, such as in a coherent statement. Additionally, coherence can imply a logical, orderly, and aesthetically consistent relationship among different parts. In the context of signal analysis, the ordinary coherence function serves as a measure of the causal relationship between two signals while considering the presence of other signals. A coherence function of one indicates a complete relationship between the two signals. If the coherence function between two input signals equals one, it signifies that the two inputs are entirely related. The coherence (C) between these two signals can be computed using the following formula:
    $$C_{ij}(f) = \frac{|S_{ij}(f)|^2}{S_{ii}(f) S_{jj}(f)},$$
    Here, $S_{ij}(f)$ represents the cross-spectral density between signals $i$ and $j$ at frequency $f$, while $S_{ii}(f)$ and $S_{jj}(f)$ represent the respective auto-spectral densities of signals $i$ and $j$ at frequency $f$.
  Phase locking value (PLV): phase synchronization between two narrow-band signals is commonly assessed using the Phase Locking Value (PLV). The PLV statistic serves as a measure of connectivity and can be considered a proxy for it. In essence, if the EEG signals in two channels (electrodes) exhibit a higher degree of synchronous rising and falling during an experimental condition compared to a baseline value, it indicates increased synchronization or connectivity between these electrodes. Conversely, if the PLV is lower than the baseline value, it suggests desynchronization or decreased connectivity between the two electrodes. It’s important to note that this metric focuses solely on the co-variation in the phase of the EEG signals and does not consider changes in the signal power between the two electrodes^57^. The PLV can be computed using the following formula:
    $$\text {PLV}_{ij} = \frac{1}{T_w} \left| \sum _{t=1}^{T_w} e^{i(\phi _i(t) - \phi _j(t))} \right| ,$$
    Here, $\phi _i(n)$ and $\phi _j(n)$ are the instantaneous phase angles of signals $i$ and $j$ at time point $n$, respectively.
  Phase lag index (PLI): the primary purpose of introducing the Phase Lag Index (PLI) is to obtain reliable estimates of phase synchronization that are not affected by common sources, such as volume conduction or active reference electrodes in the case of EEG measurements. The PLI is computed based on the asymmetry of the distribution of instantaneous phase differences between two brain regions. It operates on the principle that a consistent phase lag corresponds to a time lag between two time series. In essence, the PLI quantifies the level of synchronization between pairs of signals, indicating functional connections between different regions of the brain^58^. For example, if we consider the PLI in the theta frequency range for the connection between the left and right temporal regions, it reflects the degree of synchronization of oscillations in the 4–8 Hz range between these two regions, ranging from a lower PLI value for chaotic or unsynchronized activity to a higher PLI value for synchronized activity^59^. The PLI is calculated using the following formula:
    $$\text {PLI}_{ij} = \frac{\left| \frac{1}{T_w} \sum _{t=1}^{T_w} \text {sign}(\sin (\phi _i(t) - \phi _j(t))) \right| }{\frac{1}{T_w} \sum _{t=1}^{T_w} |\sin (\phi _i(t) - \phi _j(t))|}.$$
    Here, $\phi _i(n)$ and $\phi _j(n)$ are the instantaneous phase angles of signals $i$ and $j$ at time point $n$, respectively.

### Machine learning classification

#### The k-nearest neighbors

**Fig. 10:**
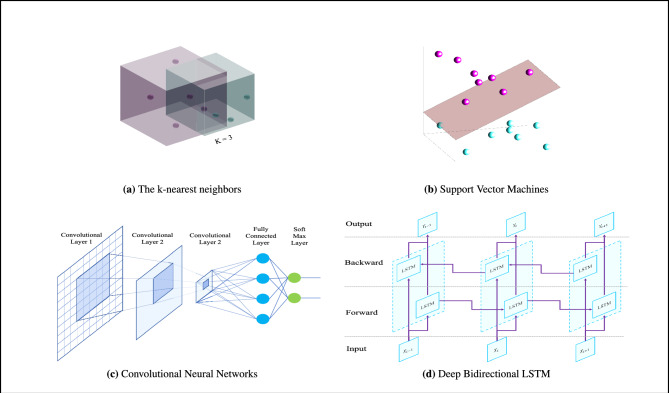
Architecture of learning methods.

The KNN is a widely used machine learning technique that has also been adapted for large-scale data mining tasks. The basic concept behind KNN is to leverage a substantial amount of training data, where each data point is defined by a set of variables. In a conceptual sense, each data point is plotted in a high-dimensional space, where each axis represents an individual variable. When presented with a new (test) data point, the goal is to identify the K nearest neighbors that are closest or most similar to it in the defined space^60^ (see the Fig. 10). To compute the k-NN classification for EEG signals, let’s assume we have a new, unlabeled EEG signal represented by the feature vector $x$. The k-NN algorithm involves the following steps:

1. Compute the distance between the feature vector of the unlabeled signal $x$ and the feature vectors of all labeled training signals.
2. Select the k nearest neighbors with the shortest distances to the unlabeled signal $x$.
3. Assign the label of the majority class among the k nearest neighbors as the predicted label for the unlabeled signal $x$.

#### Support vector machines

SVM is a supervised machine learning algorithm that is applicable for both classification and regression tasks. The fundamental model of SVMs was introduced by Cortes and Vapnik in 1995. The objective of the SVM algorithm is to identify a hyperplane in the data space that maximizes the minimum distance, known as the margin, between instances belonging to different classes within a training set. The key characteristic of SVM is that it solely relies on the support vectors, which are the data points located at the margin edges, to separate the instances, rather than relying on differences in class means. By utilizing the support vectors, the SVM algorithm defines the separating hyperplane, and hence it is named Support Vector Machines^61^ (see the Fig. 10). To apply the SVM algorithm for EEG signal classification, the following steps are undertaken:

1. Select a suitable kernel function (e.g., linear, polynomial, radial basis function) to map the input features into a higher-dimensional space.
2. Define the SVM model and optimization objective, which aims to find the hyperplane that maximizes the margin while minimizing classification errors.
3. Solve the optimization problem to obtain the optimal parameters that define the hyperplane.
4. Predict the class label of new, unlabeled EEG signals based on their position relative to the learned hyperplane.

#### Convolutional neural networks

CNNs are a type of deep learning algorithm that excel at processing and extracting features from structured data, such as images and time series data. CNNs have also been successfully applied to EEG signal analysis, offering a powerful tool for tasks like EEG-based brain-computer interfaces, sleep stage classification, and MSSTNet detection^62^. In the realm of EEG signal analysis, CNNs are specifically engineered to autonomously acquire and extract pertinent features from unprocessed EEG data. The CNN architecture encompasses essential elements such as convolutional layers, pooling layers, and fully connected layers. Convolutional layers employ filters to capture local patterns and spatial relationships within the EEG signals, while pooling layers downsample the feature maps to reduce dimensionality. Fully connected layers integrate the learned features and undertake classification or regression tasks. Consider a labeled training dataset $(x_1, y_1), (x_2, y_2), ..., (x_N, y_N)$, where $x_i$ denotes the raw EEG signal and $y_i$ denotes the corresponding class label (see the Fig. 10). We aim to formulate the application of CNNs on EEG signals. The CNN algorithm encompasses the following steps:

1. Preprocessing the raw EEG signals, which may include filtering, normalization, and segmenting into smaller time windows.
2. Constructing the CNN architecture by specifying the number and types of layers, including convolutional layers, pooling layers, and fully connected layers.
3. Training the CNN on the labeled training dataset to learn the optimal weights and parameters that minimize the classification error.
4. Evaluating the trained CNN on a separate validation dataset to assess its performance and make any necessary adjustments.
5. Using the trained CNN to predict the class labels of new, unlabeled EEG signals.

#### MSSTNet classification

In the study “Epileptic MSSTNet Classification With Symmetric and Hybrid Bilinear Models”, a hybrid deep learning framework based on bilinear models was proposed. In this method, EEG signals are first transformed into spatio–temporal representations using the Short-Time Fourier Transform (STFT). Then, a CNN is employed to capture spatial and local patterns, while an RNN is used to model long-term temporal dependencies. Subsequently, a bilinear pooling layer combines the features extracted from the CNN and RNN branches through second-order interactions, enabling the model to learn richer representations. This integration ultimately improves performance in the task of MSSTNet classification from EEG^63^.

#### Proposed a novel combined method

The analysis of EEG signals for the diagnosis of schizophrenia using machine learning methods faces inherent weaknesses. A fundamental challenge lies in effectively combining features with diverse dimensions. Traditional techniques like SVM and KNN restrict inputs to vector form, where each component represents the output size of a specific feature from a device channel. This limitation hampers the simultaneous utilization of multiple channel features, unless auxiliary methods such as PCA are employed to merge the outputs and create a vector representation.

Fortunately, methods such as CNN have successfully addressed this challenge. Within the realm of CNN, inputs can take the form of higher-dimensional matrices. For instance, users can construct a matrix where rows correspond to desired device channels and columns represent distinct features. This approach allows for an $m\times n$ matrix to serve as input, without compromising the data’s inherent properties, thereby enabling simultaneous disease diagnosis.

**Fig. 11:**
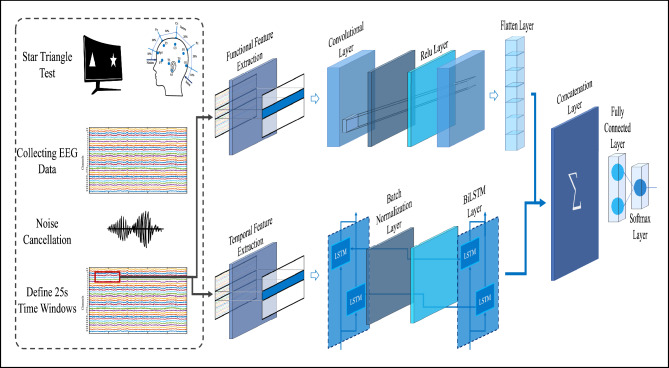
Proposed deep learning method architecture.

While CNN methods outperform SVM and KNN, their accuracy in disease diagnosis still requires improvement. Consequently, our article introduces a novel deep and hybrid learning method with several advantages, the most noteworthy being its exceptional accuracy in diagnosing diseases from EEG signals. Moreover, our method grants users the flexibility to consider inputs with different dimensions, comprising matrices of varying sizes. As exemplified in our article, we segregate temporal and functional features, resulting in matrices of dissimilar dimensions. Simultaneously considering these matrices as inputs to our proposed method enhances the diagnostic capabilities. Such versatility is not available in other methods like CNN.

By harnessing the potential of our deep and hybrid learning approach, we strive to significantly enhance the accuracy and efficacy of EEG-based schizophrenia diagnosis. The ability to simultaneously consider inputs of different dimensions, as demonstrated through the separation of temporal and functional features, holds promising implications for the field. Our work paves the way for future advancements in the analysis of EEG signals and underscores the importance of tailored methodologies to improve disease diagnosis and patient outcomes.

As mentioned in the article, the proposed method involves categorizing inputs into temporal characteristics and functional characteristics, each with different dimensions. The functional features category consists of a matrix with dimensions corresponding to the binary combinations of 31 channels (465 rows) and three columns representing PLI, PLV, and Pearson correlation. On the other hand, the temporal features category comprises a matrix with 31 rows (representing the channels) and four columns including variance, absolute energy, autoregression, and Hjorth mobility. These distinct feature types allow for the utilization of various neural network structures.

The CNN neural network is a suitable choice for disease diagnosis using functional features due to its ability to extract complex features and identify specific patterns. Therefore, it is selected to extract patterns from the functional features line. The convolutional layer is applied after the input layer, employing filters to slide over the input matrix and perform convolutional operations on its features. A Batch Normalization layer follows to normalize the output values of the preceding layers, facilitating faster and more stable network training. The Rectified Linear Activation function is then used to determine positive and negative sequences from the input values. This sequence of layers is repeated sequentially, culminating in a flatten layer that converts the two-dimensional data to one-dimensional, enabling integration with another network.

For the temporal feature line, which contains complex data with historical information, the BiLSTM neural network is well-suited. The BiLSTM network can effectively model these features by utilizing both past and future information to make informed decisions. Therefore, it is employed for the input comprising temporal features. BiLSTM is a type of recurrent neural network consisting of two LSTM layers, one forward LSTM layer and one backward LSTM layer (see the Fig. 10). These layers process the data in both forward and backward directions. Following the BiLSTM layer, a Batch Normalization layer, Rectified Linear Unit layer, and another BiLSTM layer are sequentially applied.

**Table 7:** Comparison of classifiers.

Feature	KNN	SVM	CNN	BiLSTM	MSSTNet classifier	Proposed method
Category	Traditional ML	Traditional ML	Deep learning	Deep learning	Hybrid deep learning	Hybrid deep learning
Classification Description	Nearest neighbors	The best decision boundary	Local pattern recognition	Sequential data processing	Combination of CNN and RNN	Combination of CNN and BiLSTM
Data structure	Feature vector	Feature vector	Feature tensor	Feature tensor	Combination of feature tensor	Combination of feature tensor
Key parameters	Number of neighbors (k), distance function	Type of kernel, kernel parameters, C (penalty parameter)	Number of layers, number of filters, filter size, activation function	Number of layers, number of cells, forget gate	Number of layers, number of filters, filter size, activation function, number of cells, forget gate	Number of layers, number of filters, filter size, activation function, number of cells, forget gate

To combine the features extracted from both networks, the Concatenation Layer neural network is used at the end of both input lines. This layer, a type of deep neural network layer, integrates information from multiple input layers in a successive manner. It proves particularly beneficial in deep model architectures for combining information from different sources and extracting more complex features from the input data. A Fully Connected Layer is employed after the Concatenation Layer to effectively process the combined information from different sources and make the final decision. This layer plays a crucial role in aggregating and combining features, thereby adding complexity to the model.

Finally, the Softmax Layer is utilized to select the final category based on the probabilities provided by the model, generating a meaningful output for the classification problem. This layer assists the model in making the ultimate classification decision and instills confidence in the model’s decision-making process. The architecture of the proposed deep learning method depicts in Figure 11.

In this research, an attempt has been made to conduct a comprehensive evaluation of the application and utilization of the information available in features by selecting a diverse range of classification algorithms. Each of the traditional classifiers to the up-to-date and deep classifiers has its own specific characteristics. For example, the KNN classifier only accepts one feature as input, while the CNN classifier accepts a combination of features of one type. However, The main point of the proposed method is that, the proposed method can simultaneously receive a combination of two types of functional and temporal features and, using information from both types of features, distinguish healthy individuals from patients. More information about the methods is provided in the Table 7.

## Data Availability

Data are available upon reasonable request by emailing the corresponding author.

## Code availability

The source code for feature extraction, normalization procedures, and classification methods used in this study is publicly available at https://github.com/soltanmahdiam/EEG.
